# Gut microbes in central nervous system development and related disorders

**DOI:** 10.3389/fimmu.2023.1288256

**Published:** 2024-01-26

**Authors:** Yumeng Gan, Yao Chen, Huijie Zhong, Zhuo Liu, Jiawei Geng, Huishan Wang, Wenxue Wang

**Affiliations:** ^1^ Department of Infectious Disease and Hepatic Disease, The First People's Hospital of Yunnan Province, The Affiliated Hospital of Kunming University of Science and Technology, Kunming, Yunnan, China; ^2^ School of Medicine, Kunming University of Science and Technology, Kunming, Yunnan, China; ^3^ State Key Laboratory of Genetic Resources and Evolution, Kunming Institute of Zoology, Chinese Academy of Sciences, Kunming, China; ^4^ Faculty of Life Science and Technology, Kunming University of Science and Technology, Kunming, Yunnan, China; ^5^ School of Basic Medicine, Yunnan University of Chinese Medicine, Kunming, Yunnan, China

**Keywords:** CNS development, gut microbes, autism spectrum disorder, Rett Syndrome, Angelman Syndrome

## Abstract

The association between gut microbiota and central nervous system (CNS) development has garnered significant research attention in recent years. Evidence suggests bidirectional communication between the CNS and gut microbiota through the brain-gut axis. As a long and complex process, CNS development is highly susceptible to both endogenous and exogenous factors. The gut microbiota impacts the CNS by regulating neurogenesis, myelination, glial cell function, synaptic pruning, and blood-brain barrier permeability, with implication in various CNS disorders. This review outlines the relationship between gut microbiota and stages of CNS development (prenatal and postnatal), emphasizing the integral role of gut microbes. Furthermore, the review explores the implications of gut microbiota in neurodevelopmental disorders, such as autism spectrum disorder, Rett syndrome, and Angelman syndrome, offering insights into early detection, prompt intervention, and innovative treatments.

## 1 Introduction

The mammalian central nervous system (CNS), consisting of the brain and spinal cord, exhibits unparalleled molecular, genetic, behavioral, developmental, and evolutionary complexity. A well-developed CNS is critical for survival, overseeing and directing behaviors through the transmission, storage, and processing of information. Recent research has emphasized the importance of interactions between gut microbes and the CNS, highlighting their ability to modulate host physiology, metabolism, immune function, brain function, and behavior (1). Early life represents a period of maximal bodily plasticity and heightened CNS sensitivity (2, 3). As the primary molecular interface, the intestine can influence the physiological environment during pregnancy, thus exposing the fetus to microbial signals before birth. Postnatally, the intestinal flora in infants undergoes rapid establishment and stabilization during the first two years of life, creating a persistent association between the host and commensal microbes (1, 3). The human gut microbiota exhibits remarkable genetic diversity, with over 22 million sequenced genes and an extensive library of unique enzymes capable of producing and modifying diverse chemical structures (4). Over evolutionary timeframes, microbial colonization has become integrated with CNS development programming, facilitated by a bidirectional connection between the gut and brain (4). In the prenatal phase, the fetus is exposed to microbial derivatives (e.g., metabolites, peptidoglycan) and maternal immune responses, both of which significantly influence CNS development (5, 6). Furthermore, gut microbial colonization during early postnatal life can impact CNS development and subsequent behavior (7, 8). Consequently, both prenatal and postnatal periods emerge as pivotal windows wherein gut microbes influence the CNS.

Neurodevelopmental disorders (NDDs) encompass a spectrum of chronic conditions impacting CNS functions during developmental stages, including motor skills, cognition, communication, and behavior (9, 10). Gastrointestinal (GI) comorbidities in NDDs, such as autism spectrum disorder (ASD), Rett syndrome (RTT), and Angelman syndrome (AS), exhibit a strong correlation with disease severity and pronounced symptoms of irritability, anxiety, and social withdrawal (11). These observations implicate the gut microbiome in the modulation of NDD severity and associated GI symptoms. While NDDs are usually studied from a genetic perspective, the above evidence has shifted attention toward the potential link between NDDs and gut microbiota.

In this review, the relationship between brain development and gut microbiome across various developmental stages is elaborated upon, with an emphasis on the role and mechanisms of microbes in related NDDs.

## 2 Gut microbial-brain interaction – “microbiota-brain-gut axis”

The mammalian body functions as an expansive “biochemical factory”. Beyond its native cells, it hosts trillions of microorganisms that create complex ecological niches both internally and externally (12). The gut, in particular, is a complex and dynamic ecosystem, inhabited by bacteria, archaea, viruses, and fungi (13, 14). This gut microbiota influences multiple aspects of host physiology, including nutritional balance, cellular metabolism, immune system, mucosal barrier permeability, and bidirectional communication between the gut and brain (15, 16).

**Figure 1:**
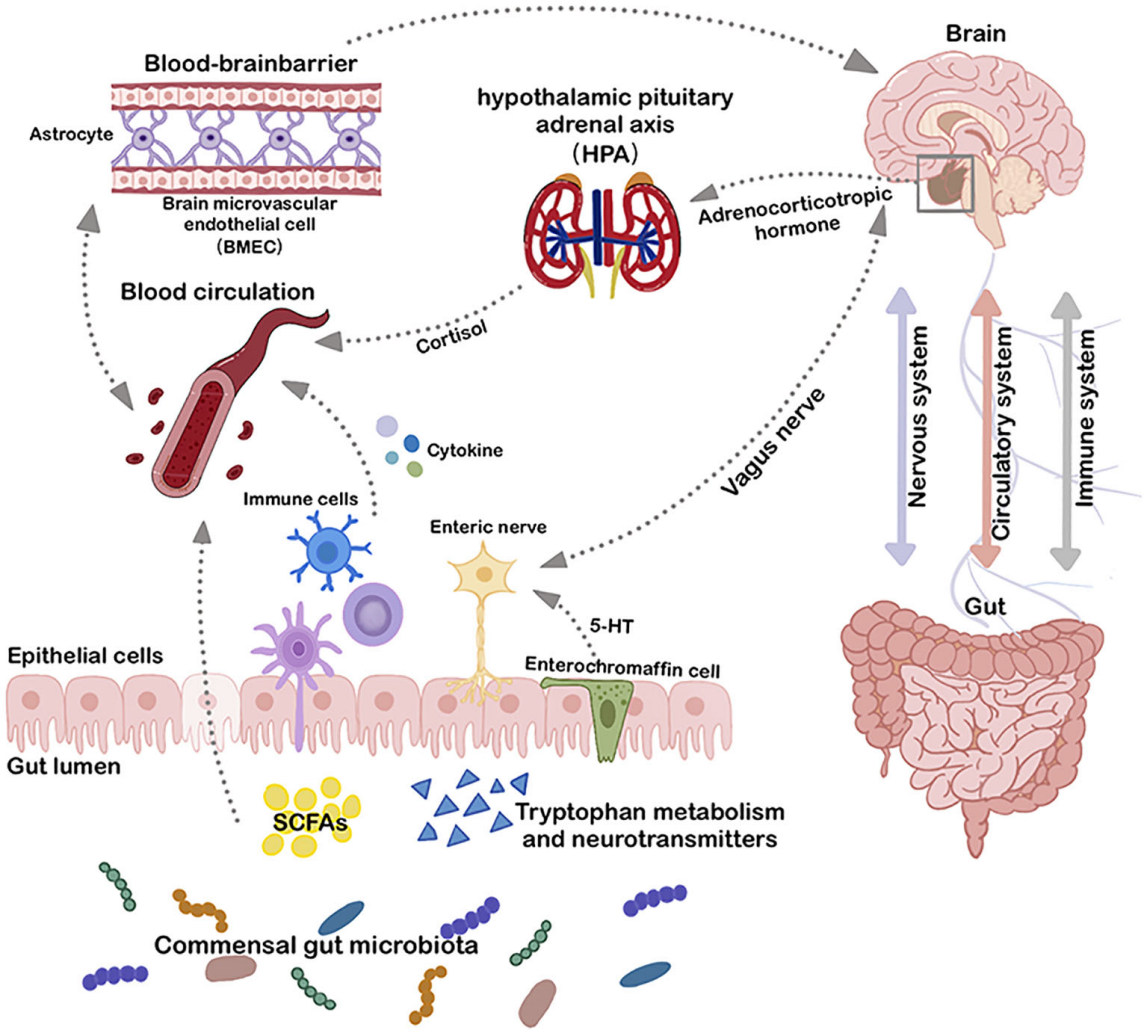
Microbiota-gut-brain axis. The bidirectional connection between the gut and the brain involves three major systems: the immune system, the circulatory system, and the nervous system. Multiple direct (e.g. vagus nerve) and indirect (e.g. short-chain fatty acids, cytokines, and key dietary amino acids, such as tryptophan) pathways exist to modulate the CNS with gut microbiota and to affect the gut microbial environment through the CNS. BMEC, brain microvascular endothelial cell; HPA, hypothalamic pituitary adrenal axis; SCFAs, short-chain fatty acids; 5-HT, 5-Hydroxytryptamine.

The gut and brain communicate bidirectionally through integrated metabolic, endocrine, neurological, and immune pathways. Key components of this system include the vagus nerve, hypothalamic-pituitary-adrenal (HPA) axis, microbial metabolites, immune mediators such as cytokines, and enteroendocrine signaling (16, 17) (**Figure 1**). This interaction can be categorized into three primary pathways: Firstly, microbial metabolites can interface with the enteric nervous system (ENS), activating the vagus nerve and subsequently communicating with the CNS (18). Secondly, metabolites produced by gut microbes may traverse the intestinal barrier, entering the circulatory system and subsequently accessing the CNS via the blood-brain barrier (BBB) to modulate its functions (19, 20). Lastly, microbial-associated molecular patterns (MAMPs) such as lipopolysaccharide (LPS) and other microbiota-produced metabolites can elicit responses from the immune system, leading to cytokine release from immune cells, which directly influence the CNS (1). The gut-brain axis is not a linear system, but a circular feedback loop that communicates via multiple pathways. The bacterial-associated factors enter the circulation and modify peripheral immune cells via blood transport. Modified peripheral immune status promotes interactions with the BBB and neurovascular units (21). This allows microbiome-induced factors, cytokines, and immune substances to cross the BBB, affecting its integrity, transport rate, and triggering the release of neuroimmune substances from barrier cells (21). This involves simultaneously the nervous system, the immune system and the circulatory system. Multiple pathways are intertwined and together channel the bidirectional communication between the gut and the CNS. Notable metabolites in this context include short-chain fatty acids (SCFAs), bile acid metabolites, nervous system agonist transmitters such as γ-aminobutyric acid (GABA), tryptophan precursors, 5-hydroxytryptamine (5-HT), and catecholamines. These metabolites, by engaging in host signaling, can influence host metabolic processes and immune responses (22–25).

Within the regulatory framework of the microbiota-gut-brain axis, the gut engages in bidirectional communication with the brain, facilitating the influence of gut microbes on CNS development and related diseases. Investigating the relationship between the gut microbiota and CNS can offer insights into CNS pathologies and new therapeutic strategies. However, several unresolved questions remain: the exact mechanisms by which gut microbes influence CNS development and associated disorders; the repercussions of CNS-related diseases on the gut and its microbial community; and the efficacy of interventions involving intestinal probiotics and prebiotics for treating CNS diseases. These topics warrant further research.

## 3 CNS development and gut microbes

### 3.1 Complex and sensitive CNS development process

**Figure 2:**
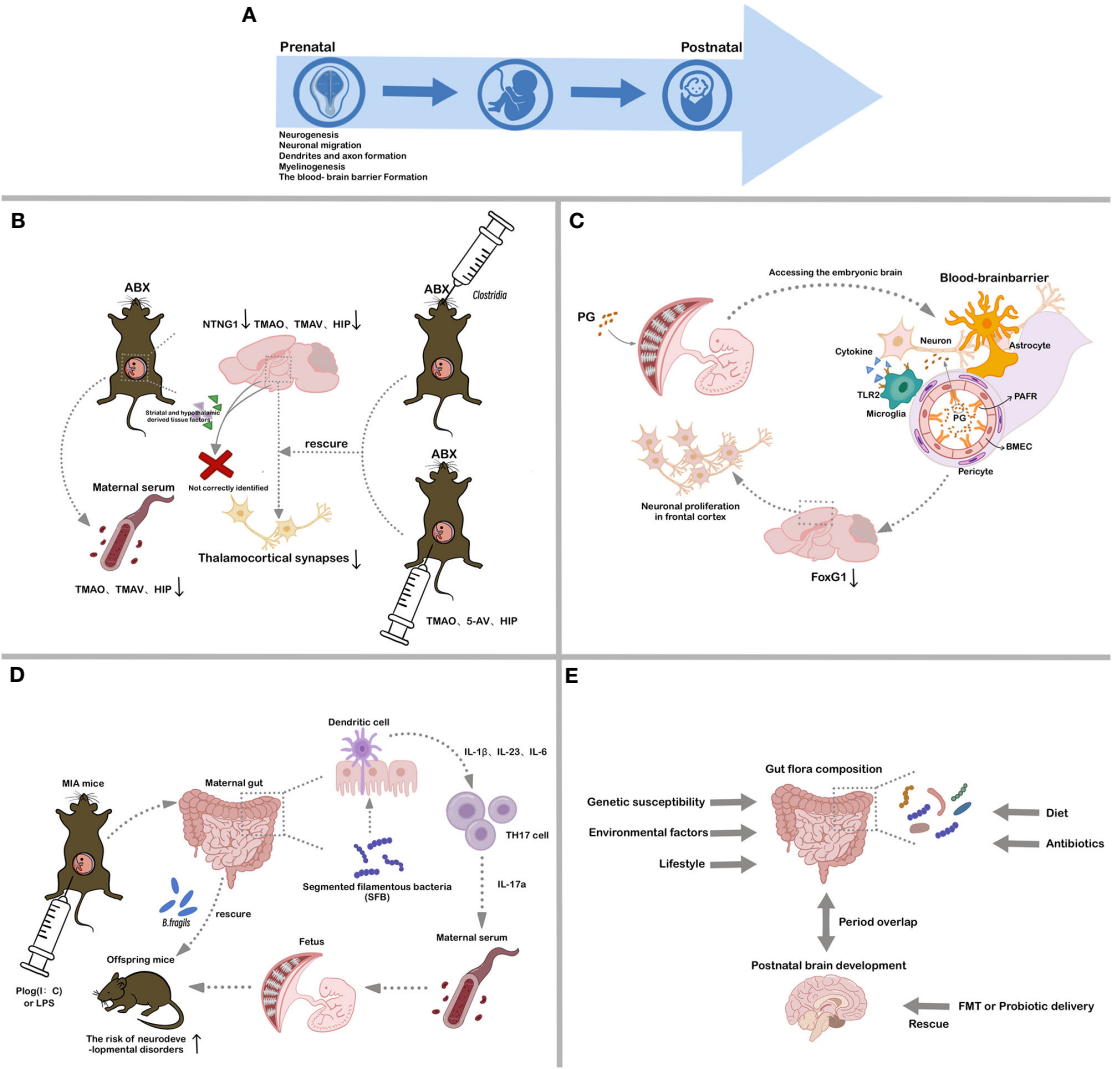
Associations between different stages of CNS development and gut microbiota. **(A)**. Critical processes in CNS development; **(B)**. Influence of maternal gut microbial metabolites on fetal brain development; **(C)**. Influence of bacterial LPS on fetal brain development; **(D)**. Influence of maternal gut microbial mediation of immune system on fetal brain development; **(E)**. Influence of establishment of gut microbes in early postnatal life on host CNS development. ABX, antibiotic-treated; TMAO, trimethylamine-N-oxide; TMAV, N, N, N-Trimethyl-5-aminovalerate; HIP, hippurate; 5-AV, 5-Aminovalerate; PG, peptidoglycan; PAFR, platelet activating factor receptor; BMEC, brain microvascular endothelial cell; MIA, maternal immune activation; SFB, segmented filamentous bacteria; FMT, fecal microbiota transplantation.

CNS development is a long and complex process that continues from the fetal stages to early adulthood (**Figure 2A**). During embryonic development, the neural plate undergoes longitudinal folding to produce the neural tube, which forms the rudimentary CNS (26). Central neurodevelopment involves neurogenesis, neuronal migration, dendritic and axonal formation, synaptogenesis, and interneuronal connection formation (27, 28). Notably, processes such as dendritic expansion, dendritic spine and synapse formation, and synaptic pruning are the most time-consuming steps in human neuronal development (29–31). As the CNS matures, the developmental trajectories of neuronal and non-neuronal cells intertwine, including events such as myelinogenesis, angiogenesis, and blood-brain barrier formation (32). While most brain neurons are formed during the fetal stages, postnatal CNS development is dominated by an increase in glial cells and neural outgrowth (33).

Given the prolonged and complex nature of CNS developmental processes, spanning both prenatal and postnatal periods, they are highly sensitive and vulnerable to internal and external environmental factors (34). Emerging evidence indicates that the gut microbiota plays a crucial role in CNS development. Germ-free (GF) mice serve as a valuable model for elucidating the effects of microbiota on CNS function and development (35, 36). Using this mouse model, key neurodevelopmental processes, including neurogenesis, cell migration, myelin formation, and microglia activation, have been linked to gut flora composition (37–40). Compared with conventional (specific pathogen-free; SPF) mice, GF mice display increased antidepressant-like behavior, risk-taking behavior, and hyperactivity, accompanied by deficits in learning and memory (22, 41, 42). Notably, these mice also show variations in the expression of 5-hydroxytryptamine receptors (5-HT 1A), synaptophysin, and neurotrophic factors (e.g., BDNF) in the hippocampus, as well as impaired blood-brain barrier functionality and increased myelin formation in the prefrontal cortex (42, 43). From a transcriptome sequencing perspective, the increase of certain immediate early response genes (e.g. Fos, Fosb, Egr2 or Nr4a1) in the GF mice amygdala is associated with increased CREB signaling, and suggests a link between the microbial establishment during early life and neurodevelopmental disorders (44). Studies have also highlighted the potential involvement of the microbiome in neurological disorders, such as depression, anxiety, schizophrenia, ASD, Alzheimer’s disease, Huntington’s disease, Parkinson’s disease, and multiple sclerosis (45, 46). Such findings underscore the pivotal contribution of the gut microbiota in CNS development and provide novel insights into the pathogenesis and therapeutic strategies for congenital CNS disorders.

### 3.2 Prenatal maternal gut microbes in offspring CNS development

#### 3.2.1 Maternal gut microbe-associated molecules in fetal brain development

The absence of fetal microbiota is commonly acknowledged. Nonetheless, the fetus is inevitably exposed to maternal-origin microbial-associated molecules, including metabolites, peptidoglycan (PG), and LPS (47–49) (**Figures 2B–D**). Various studies have established an association between maternal gut microbiota and abnormalities in offspring brain function and behavior. However, the precise mechanistic pathway by which this maternal impact shapes fetal brain development during the crucial prenatal period remains unknown.

During gestation, maternal gut microbial metabolites not only regulate maternal health but also exert effects on fetal brain development. While the molecular mechanisms underlying the effects of gut microbial metabolites on the brain remain elusive, certain metabolites, such as TMAO (Trimethylamine-N-oxide), TMAV (N, N, N-Trimethyl-5-aminovalerate), and HIP (Hippurate), have been implicated in neurological disorders and neurite growth (50) (**Figure 2B**).

Fetal brains from antibiotic-treated (ABX) dams exhibit decreased NTNG1 gene expression, which encodes the Netrin G1 protein essential for neuronal axonal growth, and fewer thalamocortical synapses relative to those from SPF dams (51–53). *In vitro* co-cultures pairing the thalamus with the striatum and hypothalamus demonstrate that thalamic neurons from ABX dams inadequately discern associated elements from the striatum and hypothalamus (53). Deficiencies in the maternal microbiome impede fetal thalamic reactions to tissue-related factors, leading to a reduction in thalamocortical synapses due to the lack of synaptic guidance signals (53). Specific microbial colonization, notably Clostridia, during pregnancy can ameliorate these alterations (53). Maternal serum and fetal cerebral concentrations of TMAO, TMAV, and HIP are diminished in ABX group relative to SPF group (53, 54). However, intraperitoneal delivery of TMAO, 5-AV (5-Aminovalerate), and HIP, or specialized gut microbe colonization in ABX dams, rescues these abnormalities, restoring fetal cerebral metabolite concentrations, thalamic synaptic densities, and tactile sensitivity in adults (53). These findings emphasize that the impact of maternal gut microbiota on fetal brain metabolic profiles and gene expression commences during the prenatal phase, underscoring the critical importance of gestation as a period during which maternal microbiota actively fosters fetal neurodevelopment.

Throughout pregnancy, maternal infections and inflammation can influence fetal health and development (55). Bacterial components produced during maternal infections can traverse the placental barrier, activating the fetal innate immune system and potentially impacting brain development and postnatal cognition (**Figure 2C**). These bacterial components are not only from maternal infection but may also be from the release of normal gut microbes (56, 57). Research has indicated that prenatal maternal exposure to PG is correlated with cognitive deficits in progeny. Notably, bacterial cell wall PG can cross the placenta, entering the developing fetal brain and inducing neuronal proliferation in the frontal cortex by increasing FOXG1 expression, a process dependent on Toll-like receptor 2 (TLR2) (58). FoxG1 is a transcription factor crucial for embryonic telencephalon development and patterning and a known spatiotemporal hub gene in the brain (59). Dysregulation of FoxG1 is associated with certain disorders, such as medulloblastoma and ASD (60, 61). These observations enhance our understanding of the link between maternal gut microbes and fetal brain and immune development, as well as the interactions between PAMPs and neuronal tissues and the discovery of novel cellular signaling pathways.

#### 3.2.2 Maternal gut microbes mediate the maternal immune system to participate in fetal brain development

The maternal immune system is strongly associated with gut microbes and fetal development (**Figure 2D**). Activation of the maternal immune system during pregnancy can impact offspring physiology, neuropathology, behavior, and microbial composition. Epidemiological studies have shown that maternal prenatal infections and inflammation can significantly increase the risk of offspring developing schizophrenia and ASD (62). Researchers have developed primate and rodent maternal immune activation (MIA) models using synthetic double-stranded RNA (polyinosinic: polycytidylic acid (poly (I:C)) or TLR ligands (e.g., LPS). Offspring from these MIA models display behavioral anomalies indicative of NDDs, including diminished social behaviors, heightened repetitive actions, and communication irregularities (63–65). Changes in the gut microbiota of these offspring can significantly influence their serum metabolomic profiles (66, 67). While segmented filamentous bacteria in the prenatal gut of MIA dams can lead to atypical behaviors in offspring, intervention with Bacteroides fragilis can ameliorate some of these adverse effects (66, 67). The presence of gut microbes, combined with pro-inflammatory signals during pregnancy, induces TH17 cells, leading to elevated maternal plasma IL-17a concentrations (67). Certain gut microbes that promote TH17 cell proliferation may thereby increase NDD risks in MIA offspring. These findings indicate that MIA models can provide valuable insights into how the gut microbiome and immune response collaboratively impact the physiology, behavior, and neuropathology of offspring.

### 3.3 Influence of early postnatal gut microbe establishment on host CNS development and behavior

The commensal microbiota in organisms emerges through increasing interactions with the environment in early life, fostering enduring interactions between host and gut microbes. Influences on gut microbiota composition include host genetic susceptibility, environmental factors, lifestyle, diet, and antibiotic and non-antibiotic drug use (68) (**Figure 2E**). The early establishment of gut microbiota coincides with a crucial period of CNS development, during which antibiotic intervention and probiotic supplementation can have profound effects on brain development, structure, and function (69).

Research has linked antibiotic use in early life and adolescence with subsequent depression and behavioral challenges (70–72). Administering non-absorbable antibiotics to adult mice over a 7-day period is sufficient to reduce anxiety-like behavior, although this effect reverts to baseline upon cessation of antibiotic use and recovery of the gut microbiome within two weeks (65). Long-term antibiotic treatment from weaning to adulthood disrupts gut microbiota structure and affects brain development and behavior in mice (72). Similarly, GF animals exhibit distinctive behaviors and developmental phenotypes compared to SPF animals (22, 41, 42). Furthermore, imbalances in gut bacteria during early brain development may heighten neurodegeneration susceptibility in later life. Probiotic interventions can modulate or mitigate specific adverse conditions in both mice and humans by enhancing specific microbial populations, either temporarily or permanently (73). Current studies have also explored the potential reconstruction of healthy gut flora through fecal microbiota transplantation (FMT) (74, 75) (**Figure 2E**). These findings highlight the strong association between the gut microbiome in early postnatal life and CNS development and behavior. Approaches that utilize gut probiotics, prebiotics, and gut flora restoration are emerging as promising strategies for CNS disease treatment.

## 4 Gut microbes in NDDs

### 4.1 ASD and gut microbes

ASD represents a cluster of complex heterogeneous NDDs. The primary clinical manifestations of ASD include impaired social interactions, disordered language development, and restricted repetitive behaviors (76). Epidemiological studies indicate an increasing incidence of ASD in recent years. The World Health Organization (WHO) estimates a global ASD prevalence of about 1%, with a recent survey estimating a prevalence of 1%–5% in developed countries, potentially associated with changes in diagnostic criteria, improvements in screening and diagnostic tools, and increased public awareness (77, 78). The etiology and pathogenesis of ASD are complex, with contributions from genetic, epigenetic, and environmental factors. Environmental factors may increase the incidence of ASD by increasing genetic susceptibility (76). While the interval from prenatal and early postnatal life is considered critical for environmental impacts on ASD, the precise timing and mechanisms of action of these factors are yet to be fully elucidated.

**Figure 3:**
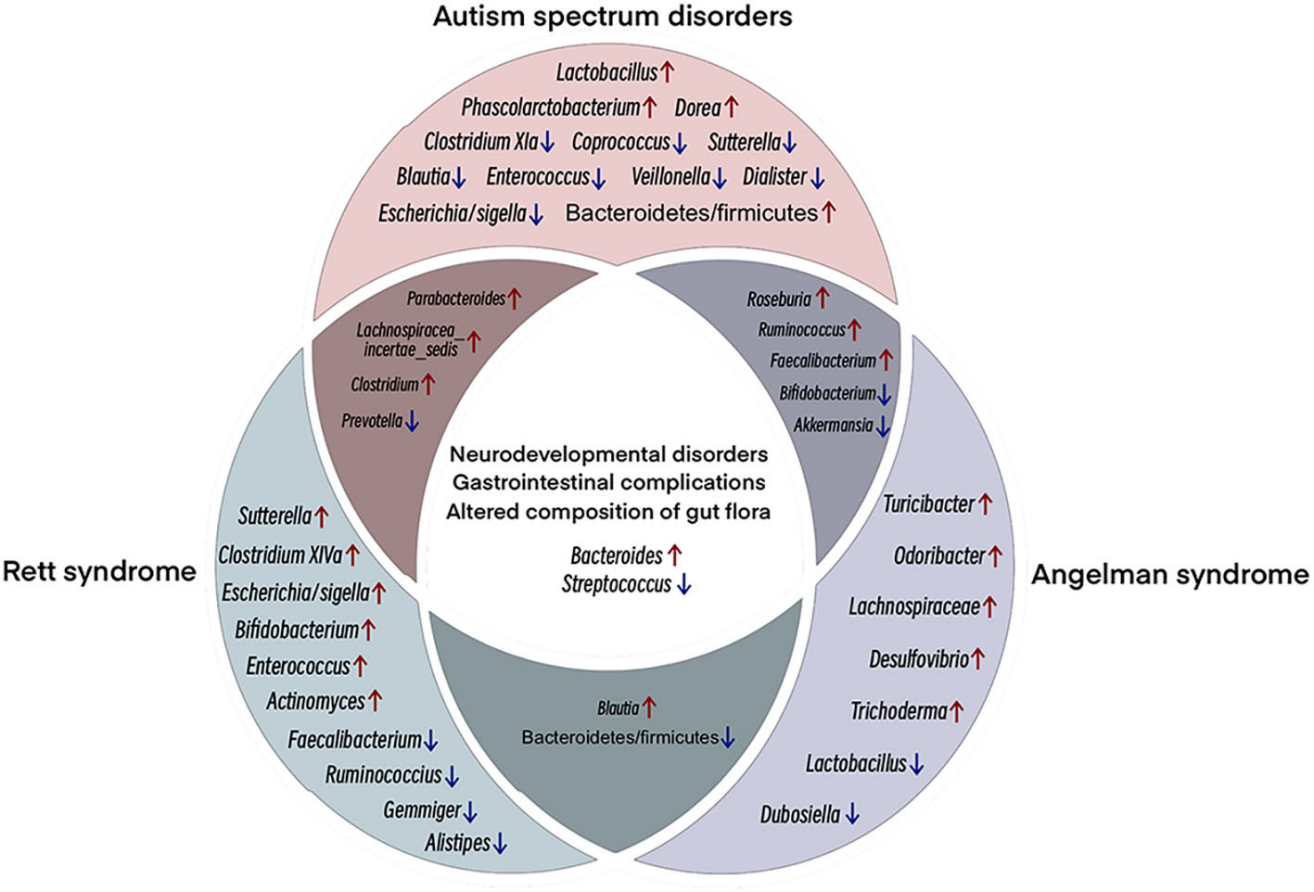
Venn diagram showing shared clinical and microbial features among ASD, RTT, and AS. NDDs with similar clinical features show some microbial compositional features that are enriched/reduced in the same taxa. The main overlap, both clinical and microbial. Among the three NDDs, ASD, RTT and AS, multiple studies have shown increased abundance of *Bacteroides* and decreased abundance of *Streptococcus*. Different colored circles represent different NDDs; pink, ASD; green, RTT; purple, AS. Overlapping regions have the same abundance trend. Red arrows, increasing relative abundance, blue arrows, decreasing relative abundance.

Studies have shown that GI symptoms are strongly associated with autism severity, irritability, anxiety, and social withdrawal (79, 80). Interestingly, in addition to genetic differences, ASD patients also exhibit significant differences in gut bacterial species and abundance compared to typically developing (TD) individuals (81). Such findings have triggered further research on the link between ASD and gut microbiota as well as NDDs and gut dysbiosis. At the phylum level, the gut microbiota in ASD patients predominantly consists of Bacteroidetes, Firmicutes, and Actinobacteria, and Tenericutes (82), with a higher ratio of Bacteroidetes to Firmicutes in ASD than in TD (82, 83). At the genus level, ASD individuals exhibit higher levels of *Bacteroides*, *Parabacteroides*, *Dorea*, *Phascolarctobacterium*, *Clostridium*, *Faecalibacterium*, *Ruminococcus*, *Lachnospiracea incertae sedis*, *Roseburia*, and *Lactobacillus*, but lower relative abundances of *Bifidobacterium*, *Coprococcus*, *Blautia*, *Veillonella*, *Dialister*, *Escherichia/Shigella*, *Prevotella*, *Clostridium XIVa*, *Streptococcus*, *Akkermansia*, *Sutterella*, and *Enterococcus* (82–85) (**Figure 3**). The gut flora in ASD rodent models shows similar significant differences relative to normal rodents (81).

Dysbiosis in ASD-associated gut microbiota is characterized by an overabundance of pathogenic bacteria and a reduced presence of beneficial bacteria. Research indicates that GF mice receiving FMT from ASD mice display decreased communication and increased repetitive behaviors compared to GF mice receiving FMT from TD mice (81, 86). Treating ASD mice with *Bacteroides fragilis* has been shown to correct intestinal mucosal barrier defects and improve ASD symptoms, such as stereotyped behavior and anxiety (66, 87). Certain metabolites from the order Clostridiales are correlated with repetitive behaviors and GI issues in ASD, which can be reversed following antibiotic use (88). Elevated concentrations of propionic acid (PPA), primarily produced by the phylum Bacteroidetes, are recognized as significant neurotoxic SCFAs (89). SCFAs, when transported to the brain via the circulatory system, influence brain development by modulating serotonin and dopamine synthesis (90). Both animal models and clinical trials involving probiotics, such as *Lactobacillus* and *Bifidobacterium*, report improvements in mood, anxiety, sleep quality, and depression (91, 92). These findings further underscore the correlation between gut flora and neuropsychiatric and behavioral shifts in affected individuals.

### 4.2 RTT and gut microbes

Methyl-CpG binding protein 2 (MECP2) is an important gene in the pathogenesis of ASD and RTT and critical for regulating synaptic activity in early postnatal life (93). RTT is a progressive neurological disorder primarily caused by mutations in the MeCP2 gene on the X chromosome. GI dysfunction and constipation are commonly observed in RTT patients, suggesting a link between neurological abnormalities with intestinal function and gut microbiota (94). Research has documented structural alterations in the gut microbiome of human RTT subjects and MECP2-mutated animal models, characterized by diminished abundance and diversity (95, 96). At the phylum level, while Actinobacteria predominates in RTT and Firmicutes predominates in TD, RTT exhibits an increased relative abundance of Actinobacteria and Firmicutes, a reduced presence of Bacteroidetes, and a notably elevated Firmicutes/Bacteroidetes ratio, indicating gut ecological dysregulation associated with RTT (95, 96). At the genus level, *Bacteroides*, *Bifidobacterium*, *Parabacteroides*, *Lachnospiracea incertae sedis*, *Blautia*, *Escherichia/Shigella*, *Actinomyces*, *Clostridium XIVa*, *Enterococcus*, *Clostridium*, and *Sutterella* show an increase in relative abundance in RTT, but a decrease in *Faecalibacterium*, *Streptococcus*, *Ruminococcus*, *Prevotella*, *Gemmiger*, and *Alistipes* (94, 95, 97) (**Figure 3**) Functional disruptions in MeCP2 modify the gut microbial community structure, which, in turn, alters SCFA production and gastrointestinal pathophysiology in RTT, leading to constipation, inflammation, and host cytokine dysregulation (95). This impairment in MeCP2, mediated through the gut-brain axis, manifests as a dysregulated gut ecology in RTT patients.

RTT primarily affects females, with males harboring the MECP2 mutation tending to succumb shortly after birth. MECP2 is widely expressed in mammals, predominantly in the nervous system, with higher expression in neurons than in glial cells (93). Studies have shown that male ASD patients exhibit diminished MECP2 expression in brain astrocytes, concomitant with high methylation of the MECP2 promoter in the frontal cortex, alongside the genetic mutation observed in RTT patients (98, 99). Such evidence suggests that MECP2 DNA hypermethylation in astrocytes may underlie ASD pathogenesis in males. Several studies have shown that reintroducing MECP2 expression in astrocytes of MECP2-deficient mice can ameliorate certain behavioral and molecular abnormalities (100). In Mecp2-deficient mice, increased glutamine uptake by microglia can induce toxic effects due to glutamate overproduction in hippocampal neurons, leading to dendritic and synaptic damage (101, 102). Notably, glutamate, an excitatory neurotransmitter, is highly susceptible to gut microbes and can be metabolized by bacterial glutamate decarboxylase to produce gamma-aminobutyric acid (GABA). Subsequent research has shown that GABA-producing bacteria can reduce depression- and anxiety-like behaviors in mouse models (103, 104). Exploration into RTT immune dysfunction using targeted glial cell activation has also revealed that LPS stimulation can induce a dramatic increase in mixed astrocyte-microglia pro-inflammatory cytokine release in MECP2-deficient mice (105). These findings offer promising avenues for RTT treatment, potentially ameliorating physiological functions in patients or influencing disease progression through gut flora modulation.

Cholesterol is an important component of the brain, involved in membrane transport, signal transduction, myelin formation, dendritic remodeling, neuropeptide formation, and synaptogenesis (106). While evidence suggests that nascent neurons autonomously synthesize cholesterol, this capability diminishes with development, leading to a reliance on astrocytes for cholesterol production (107). This down-regulation is absent in RTT brains, leading to lipid accumulation and metabolic dysregulation (108). Intestinal microbes maintain cholesterol homeostasis in the body, playing a key role in bile acid (cholesterol derivative) metabolism and the conversion of cholesterol into distinct metabolites through dehydrogenase activity encoded by the IsmA gene (109). Previous studies have suggested that fecal metabolites are altered in Mecp2-mutated females and associated with lipid defects in the brain (108). Recent studies have applied RTT mouse models to investigate the role of the gut microbiome and metabolome perturbations in RTT disease progression.

Glial cell lesions and dysregulated brain lipid metabolism due to MECP2 dysfunction have been implicated as primary drivers of RTT and ASD pathogenesis (110, 111). Gut microbes play a critical role in RTT disease progression, which warrants further study. Changes in the intestinal microbiota of RTT patients may reflect clinical presentations and potentially impact disease evolution. A future research challenge is the restoration of intestinal flora or colonization with specific probiotics as a novel approach for RTT management.

### 4.3 AS and gut microbes

AS is a rare genetic neurodevelopmental syndrome resulting from the gene expression deletion of maternally inherited ubiquitin ligase E3A (UBE3A) in brain neurons (112). While paternally inherited UBE3A is expressed in most peripheral organs, it is silenced in the CNS by long non-coding antisense transcripts (UBE3A-ATS) due to brain-specific imprinting. Thus, deletion of maternally inherited UBE3A can lead to complete loss of UBE3A expression in the brain (113). Clinical manifestations of AS include microcephaly, severe developmental delays, expressive communication deficits, distinct facial features, motor and coordination deficits, hypotonia, generalized epilepsy, and sleep disturbances (114). Beyond neurodevelopmental effects, GI issues are also frequently reported in AS patients (115). The gut microbiome is critical for the establishment of GI physiology and function, and alterations in microbial colonization are common in NDDs. At the phylum level, the gut microbiota of AS animal models shows an increased abundance of Bacteroidetes and Actinobacteria and a decreased abundance of Firmicutes compared to wild-type (WT) controls, leading to a lower Bacteroidetes to Firmicutes ratio in AS (116). At the genus level, AS animal models exhibit a decreased abundance of *Lactobacillus*, *Bifidobacterium*, *Dubosiella*, *Streptococcus*, and *Akkermansia* compared to WT controls, but increased levels of *Bacteroides*, *Trichoderma*, *Lachnospiraceae*, *Desulfovibrio*, *Odoribacter*, *Faecalibacterium*, *Roseburia*, *Blautia*, *Ruminococcus*, and *Turicibacter* (116) (**Figure 3**). Notably, *Lactobacillus* is associated with several diseases, including major depressive disorder and non-alcoholic fatty liver disease (117), while *Desulfovibrio* is associated with Parkinson’s disease, with its abundance in the gut directly related to disease severity (118). Furthermore, *Odoribacter* is associated with attention deficit and hyperactivity disorders, and can disrupt dopamine and serotonin levels in the gut (119). The changes observed in the AS gut microbiome are similar to those detected in the gut flora of other NDDs.

Most AS patients exhibit symptoms of impaired coordination, imbalance, and gait ataxia (120). PIEZO2, a mechanosensitive ion channel, is necessary for maintaining coordination and balance (121). Reduced PIEZO3 activity has been identified in sensory neurons from UBE3A-deficient mice and stem cell-derived neurons from AS patients. Linoleic acid (LA)-rich diets have been shown to increase PIEZO2 activity and mechanical excitability in male AS mice, ameliorating gait abnormalities. Furthermore, LA supplementation has been shown to increase PIEZO2 function in sensory neurons from UBE3A-deficient mice and in stem cell-derived neurons from AS patients (119). The gut microbiota has the capacity to metabolize lipids into bioactive metabolites (121). Recent studies have highlighted that LA-derived microbial metabolites can influence host lipid metabolism via the augmentation of peroxisomal β-oxidative metabolism (121). These metabolites also improve CNS self-immunity in multiple sclerosis mouse models, as evidenced by improved gut barrier function, attenuated inflammation, and increased in intestinal myeloid-derived suppressor-like cells (122). Strategic modulation of the gut microbiome may alleviate neurological and gastric symptoms in AS patients, thereby enhancing their overall quality of life.

Interestingly, NDDs with similar clinical features often exhibit microbial compositional similarities with enrichment or reduction in the same taxa (82–85, 94, 95, 97, 116). The main overlap, both clinical and microbial (**Figure 3**). The figure considers only the microbial changes reported in most studies (i.e., discarding inconsistent results). *Bacteroides* enrichment is frequently reported in the reviewed NDDs (82–85, 94, 95, 97, 116). This taxon, together with *Clostridium*, possesses remarkable proteolytic ability, leading to potentially toxic compounds that can disrupt gut homeostasis and permeability and affect the survival of other beneficial microorganisms, such as lactic bacteria (123, 124). Conversely, a decrease in the relative abundance of *Streptococcus*, including *Streptococcus thermophilus*, is common among NDD patients. While *Streptococcus thermophilus* is known to ferment lactose and sucrose (125), the overall relationship between *Streptococcus* and GI health remains largely unexplored. Remarkably, although *Streptococcus* species are generally non-pathogenic, some strains are associated with multiple metabolic disorders (126). *Streptococcus thermophilus* can increase the abundance of gut probiotics, including *Bifidobacterium* and *Lactobacillus*, via β-galactosidase (127). Predominant shifts in gut microbial communities can induce an increase of pro-inflammatory species and a decrease in probiotic species, which may promote alterations in gut permeability and barrier functionality.

Alterations in gut flora are associated with NDDs (e.g., ASD, RTT, AS) and GI disorders, highlighting the important role of gut microbes in gut-brain communication (11, 79, 80, 94, 115, 116). Whether these shifts in gut flora cause GI symptoms in NDDs and influence disease progression remains unclear. Based on current findings, evaluating gut flora alterations is necessary to study the effects of gut microbiota on GI physiology and motor behavior in NDD patients and related animal models.

## 5 Summary and future directions

In summary, the gut microbiota is closely linked to CNS development and related diseases. Gut microbes and their metabolites play a crucial role in brain-gut axis interactions. While exploration of the gut-brain axis remains at an early stage, certain basic circuits have begun to emerge and specific neurodevelopmental pathways may require a response from gut microbial signals. Mammalian CNS development is a long process that begins during pregnancy with the differentiation of neural progenitor cells and extends into late adolescence, potentially persisting across the whole life cycle. These developmental processes entail relevant gene expression and environmental inputs, with disruptions in any of these events fundamentally altering neural outcomes (128). Functioning as an environmental determinant, the gut microbiome is highly likely to exert an enduring influence on the structure and functionality of the CNS (129). Many studies have indicated that changes in the maternal gut microbiota can modulate offspring gut microbiota, neurodevelopment, and behavior (130). In humans and rodents, perinatal administration of antibiotics can affect the health and immune status of offspring (131). In mouse models, maternal exposure to antibiotics alters gut microbiota and reduces motility in dams and offspring, with anxiety-like behavior and motor deficits in the latter (132). As previously discusses, important processes (e.g., synaptic growth and neuronal proliferation) in fetal CNS development are affected by maternal microbial-associated molecules (e.g., metabolites, PG, and LPS), with maternal gut microbes contributing to fetal brain development through the maternal immune system. Furthermore, the establishment of gut microbiota in early postnatal life can influence host CNS development and behavior. These examples underscore the critical role of the gut microbiome in both prenatal and postnatal CNS development. Currently, the connection between gut microbes and the pathogenesis in CNS diseases is progressively gaining prominence as a frontier of scientific research. NDDs (e.g., ASD, RTT, AS) can disrupt gut microbial balance, potentially aggravating disease progression. By modulating gut microorganisms, it may be possible to attenuate CNS disease development and enhance host immunity, thereby ensuring human and animal well-being. Nonetheless, current clinical data regarding probiotic supplementation for disease treatment remain limited, with a lack of standardized probiotic protocols. Consequently, in-depth study of the regulatory mechanisms underpinning CNS diseases and gut microbiota holds profound implications not only for advancing gut health promotion but also for expanding the scope of CNS-related disease prevention.

## Author contributions

YG: Conceptualization, Validation, Visualization, Writing – original draft, Writing – review & editing. YC: Investigation, Validation, Writing – review & editing. HZ: Validation, Writing – review & editing. ZL: Validation, Writing – review & editing. JG: Conceptualization, Funding acquisition, Supervision, Validation, Writing – review & editing. HW: Conceptualization, Funding acquisition, Investigation, Supervision, Validation, Writing – review & editing. WW: Conceptualization, Funding acquisition, Investigation, Supervision, Validation, Writing – original draft, Writing – review & editing.
